# Factors influencing maternal death surveillance and review implementation in Dodoma City, Tanzania. A qualitative case study

**DOI:** 10.1002/lrh2.10390

**Published:** 2023-09-04

**Authors:** Nelson M. Rumbeli, Furaha August, Valeria Silvestri, Nathanael Sirili

**Affiliations:** ^1^ Department of Epidemiology and Biostatistics Muhimbili University of Health and Allied Sciences Dar es Salaam Tanzania; ^2^ Department of Obstetrics and Gynecology Muhimbili University of Health and Allied Sciences Dar es Salaam Tanzania; ^3^ Department of Parasitology and Medical Entomology Muhimbili University of Health and Applied Science MUHAS Dar es Salaam Tanzania; ^4^ Department of Development Studies, School of Public Health and Social Sciences Muhimbili University of Health and Allied Sciences Dar es Salaam Tanzania

**Keywords:** implementation, maternal death, maternal death review, maternal death surveillance and response strategy, Sub‐Saharan countries

## Abstract

**Background:**

With 295 000 maternal deaths in 2017, 94% in low‐ and middle‐income countries, maternal death is a matter of global public health concern. To address it, Maternal Death Surveillance and Response (MDSR) strategy was introduced in 2013 by the World Health Organization. With a reported maternal mortality ratio of 556:100000 per live births, Tanzania adopted the strategy in 2015. Studies are needed to understand factors influencing the implementation of MDSR in this specific setting.

**Aims and Objectives:**

The study aimed to assess the processes influencing MDSR implementation in Dodoma city council.

**Methods:**

A qualitative case study was conceptualized according to the Consolidated Framework for Implementation Research, focusing on implementation process domain. Members of MDSR committees were enrolled by purposeful sampling in the five health centres in Dodoma where the strategy was fully implemented and functional. In‐depth interviews were conducted with key informants concerning the implementation processes influencing MDSR. Saturation was reached with the 15th respondent. Qualitative inductive content analysis was used to analyse data.

**Results:**

The inclusiveness in participatory planning process, stakeholders’ readiness and accountability and collective learning were acknowledged as factors positively influencing the implementation of MDSR strategy by respondents. The interaction and alignment of influential factors were essential for successful implementation.

**Conclusions:**

MDSR implementation is positively influenced by factors that interact and converge in the building of a learning health system, to increase knowledge through practice and improve practice through knowledge. Further studies are needed to analyse the influence of additional factors at different levels of implementation to fully understand and empower the MDSR implementation network, and to better target the goal of closing the knowledge to practice loop.

**Abbreviations**
CFIR: consolidated framework for implementation research
MDG: millennium development goals
MDR: maternal death review
MDSR: maternal death surveillance and response

## 1 INTRODUCTION

According to World Health Organization (WHO) definition, maternal death is ‘*the death of a woman while pregnant or within 42 days of the termination of pregnancy irrespective of the duration and site of the pregnancy, from any cause related to or aggravated by the pregnancy or its management, but not from accidental or incidental causes*’.^1^

Maternal mortality is still an issue of global public health concern; according to WHO data about 295 000 maternal deaths occurred in 2017, the majority in low‐resource countries, where one out of every 41 pregnant women dies every day due to pregnancy issues.^2^, ^3^

In Tanzania maternal death is still challenging with the stagnation of the number of maternal mortality ratio with 556 per 100 000 live births despite different interventions.^4^, ^5^

The Maternal Death Surveillance and Response (MDSR) system is a strategy defined in the context of Millennium Development Goals (MDGs) by WHO, which consists of a continuous cycle of notification, review, analysis and response to maternal death events. Its aim is to reduce preventable maternal mortality by involving all stakeholders in the process of identifying maternal deaths, understanding their cause and planning interventions to prevent the occurrence of similar deaths in the future. The strategy has become widely established globally, especially since the publication of detailed technical guidance by WHO in 2013.^2^ However, notwithstanding the wide global uptake of the intervention, different studies have reported on the inadequate implementation of MDSR in different settings, due to the lack of implementation of the recommendations from maternal death review (MDR) sessions, the absence of verbal autopsy for deaths, the low commitment of districts,^6^ the poor availability of reports, incomplete data and missing key information.^7^ Among positive factors, planning has been identified as a catalyst for MDSR by previous studies, but the mechanism of its positive influence has not been studied.^8^, ^9^

Tanzania introduced the MDSR system in 2015 in accordance with WHO recommendations, defining specific guidelines for the collection of information from medical records, healthcare providers and family of the deceased, for summary of data and meeting discussion and, at last, for the definition of action points by the reporting committee to be sent to the Ministry of Health that will act accordingly at local and national levels to prevent future maternal deaths.^7^

Previous studies have analysed different aspects of the implementation of the MDSR in Tanzania. However, an exhaustive description of modifiable and non‐modifiable factors favouring or impairing implementation of the strategy in Tanzanian context was not well explained.^7^, ^10^ As reported for other national settings, further studies are needed to analyse in detail the influence of the constructs of the implementation process domain on the implementation of the MDSR strategy in Tanzania.

## 2 AIM AND OBJECTIVES

The aim of this qualitative study is to provide information on how the constructs of the implementation process domain, specifically planning, engagement of appropriate individuals, surveillance and response and reflection and evaluation, are considered to be of influence in the implementation of MDSR by members of the MDR in Dodoma City Council.

## 3 METHODS

### 3.1 Study design, theoretical framework and methodological orientation

A qualitative study was designed and conceptualized according to Consolidated Framework for Implementation Research (CFIR), specifically focusing on the implementation process domain, which explores strategies or tactics that might influence implementation.^11^ Qualitative in‐depth interviews with members of the MDSR committee were conducted to analyse the respondent's perspective on which implementation domain processes had influence on MDSR and how it influenced it. The use of CFRI provided a comprehensive framework and a standard taxonomy, to guide in the analysis of rich qualitative data and to facilitate the assessment of the relevance of research findings.^11^

### 3.2 Research team and reflexivity

The interviews were conducted by the principal investigator, as original research for the Master of Public Health and Implementation Science programme, under the supervision of mentors experienced in qualitative research during all phases of project implementation.

No relation was reported between the principal investigator that conducted the interviews and study participants, nor between participants and other research members.

### 3.3 Reliability of data

Trustworthiness was ensured by enhancing credibility, transferability, replicability and confirmability of results, as for gold standard in qualitative research.^12^ Accordingly, a pre‐test of the data collection tools was performed, to understand how well questions elicited responses that fulfilled the study objectives among the enrolled study participants.^12^ Additionally, peer debriefing was also organized during implementation of the project, and field notes taken during interview by the principal investigator were discussed.^13^, ^14^ Credibility was enhanced by triangulation of information from different participants to get rich information from their individual experiences regarding the research question. Replicability, transferability and confirmability of the study were ensured by following the CFIR standardized framework.^11^

### 3.4 Study setting

The study was conducted between May and June 2022, in five among the 50 Health facilities available in Dodoma city council. The Dodoma City Council bordered on the east by Chamwino district and on the west by Baha district, has a population of 559 780, 102 healthcare facilities and a total of 50 health centres.

### 3.5 Inclusion and exclusion criteria, sampling and sample size

Purposeful sampling strategy was used to select those five centres among an overall number of 50 health centres in Dodoma city. The selected facilities were the ones in which the MDSR was known to be implemented and fully functional. In each centre three participants were purposely selected, according to their availability on the day of the interview and likeliness of them being informative, due to documented engagement and the length of their working experience in the MDSR committee (> than one year).

The sample size for responders to in‐depth interviews (IDI) was of 15, which allowed to reach the saturation of themes related to the study aims, as confirmed by inter‐peer discussion among research members.

### 3.6 Methodology of approach, data collection tools and technique

Information was collected using an IDI guide, to generate data rich enough to enable analysis that went beyond surface‐level responses.^15^, ^16^

The interview topic guide consisted of 15 questions designed to guide the respondent in the description of the planning, of the engaging and of the reflecting and evaluation constructs of the implementation domain and in the description of their perceived influence on the implementation of MDSR strategy among MDSR committees (Table 1).

**TABLE 1:** Research questions and probe questions.

	Research questions	Probe questions
1	How does planning influence MDSR implementation among MDSR committees in Dodoma?	Probing questions What do you understand by the term planning? Why is planning important for MDSR implementation? How do you prepare your plans in addressing maternal death in your health facility? How do plans address MDSR in your health facility? How do you implement plans that are aimed to prevent maternal death in your health facility?
2	How engagement of appropriate stakeholders influences the implementation of MDSR among members of the MDSR committee in Dodoma?	Probing questions How do you understand the term stakeholders’ engagement? Which stakeholders do you engage in your health facilities in addressing MDSR? How do you identify your stakeholders for implementation of maternal death review in your health facility? What is the importance of engaging different stakeholders in MDSR?
3	How reflectivity is influencing the implementation of MDSR among members of MDSR committees in Dodoma?	Probing questions What information do you collect in the implementation of MDSR? How do you assess the progress of implementation of MDSR in healthcare facility? What challenges do you encounter during evaluation of implementation of MDSR in healthcare facility?

Interviews took place in Swahili language in a private location selected by the respondents among the available rooms in the Health Centre to ensure confidentiality. An average of 30 min was allocated for each interview. Permission was obtained from the responders to audio‐record the interviews.

### 3.7 Analysis of transcripts

The audio‐recorded interviews were transcribed verbatim and then translated from Swahili to English by the bilingual principal investigator. Transcripts were analysed together with field notes, and the meaning unit was extrapolated with the guidance of the CFIR code book and interview guide, using a qualitative content analysis approach. From each meaning unit data codes were opened^17^ and grouping of similar codes was done to establish subcategories using the qualitative analysis software NVIVO, version 12. Similar subcategories were then abstracted according to generate categories, that were finally used to describe the implementation processes that are influencing MDR committees in implementing MDSR strategy.

### 3.8 Reports of results

Results were described accordingly to the consolidated criteria for reporting qualitative research (COREQ) to ensure consistency.^18^

### 3.9 Ethics approval and consent to participate

The project received approval from the Muhimbili University of Health and Allied Sciences Institutional Review Board (MUHAS‐REC‐04‐2022‐1128). Informed consent was acquired from respondents and confidentiality was ensured. The procedures followed were in accordance with the ethical standards of the Helsinki Declaration (1964, amended most recently in 2008) of the World Medical Association.

## 4 RESULTS

Of the eligible participants, none refused consent, and no drop‐out was reported during the study implementation. A total of 15 respondents were enrolled (10 women and five men), including two assistant nursing officers, five nursing officers, one clinical officer, two assistant medical officers and five medical doctors.

**FIGURE 1:**
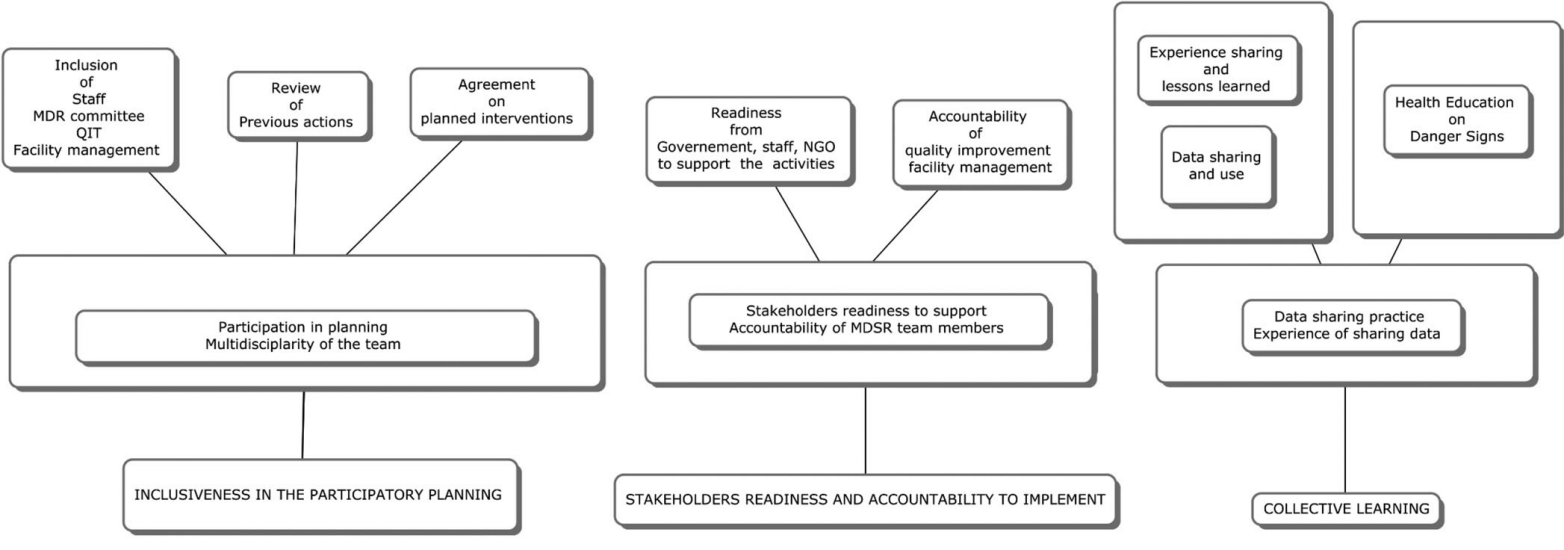
Coding process. Three categories were obtained by transcript analysis. Inclusiveness in the participatory planning; stakeholders’ readiness and accountability to implement; collective learning.

The coding process identified three major categories: (1) inclusiveness in the participatory planning process; (2) stakeholders’ readiness and accountability to implement; (3) collecting learning on implementing MDSR. The coding process was summarized in a flow chart in Figure 1.

### 4.1 Inclusiveness in participatory planning process

The majority of the participants reported that they were involved in the analysis of the state of implementation of previous plans and in further planning of activities related to MDSR on a day‐to‐day basis. Respondents perceived participatory planning as a positive factor for MDSR implementation, because of the feedback on the state of the events occurred to each of the members and an opportunity to review what wrong and plan change. Responder 09 stated:

‘Every Wednesday of the week we have a meeting where all members of MDSR participate that gives us feedback on what happened during the week. In case of the report of a maternal death, this challenges us to review where we went wrong and what we should plan so that in the future we can have good results’.

The inclusion of managers and staff of the facility in the MDSR committee was considered a support towards implementation, facilitating the satisfaction of different needs and the provision of services in a timely manner; additionally, the inclusion of staff and managers in the meetings makes every member of the facility aware of the strategy for implementation. Responder 03 quoted:

‘Inclusion of staff and management helps us to plan and prepare ourselves for providing services to clients. You find that everything is available for example drugs, infrastructure, surgical instruments, you don't start to worry’.

Participants reported multidisciplinarity of the team as the way to achieve improvement. Respondent 02 observed that:

‘MDSR committee, facility management, and staff, we have frequent participatory meetings […] if for example this time we want to improve the way we manage our clients, then we improve the plan together in a multidisciplinary way, because we have a guideline which encourages us to meet with experts of other disciplines and we are supported by facility management’.

Periodic regular planning was considered a positive factor that facilitated the attribution of tasks. According to Respondent 14:

‘We do planning as a team in order to come up with which activity needs to be done in order to implement MDSR to prevent maternal death for instance monitoring the progress of labour and normally we meet on Wednesday and we assign people responsible for performing the task’.

### 4.2 Stakeholders readiness and accountability

The importance of the engagement of stakeholders like community leaders and community healthcare workers (HCWs) was emphasized, specifically for educational intervention targeting the community. As Respondent 05 said:

‘We continue providing health education in collaboration with ward executive committee and when we are invited we normally go in those villages and provide education on different issues related to maternal health, we involve the leaders and we also cooperate with community health workers in providing education to us’.

In addition to the involvement of stakeholders directly involved in MDSR, the incorporation of the activities in facility routine plans, in the form of available checklists, resulting in the sharing of responsibility among all members of the facility was considered helpful to conduct internal audit and to ensure correct implementation. As stated by Respondent 07:

‘We are conducting internal audit using developed checklist in all of the department in this facilities and dispensaries in cascading to ensure all supplies and commodities like safe blood are available if emergencies occur services should be provided without delay’.

Synergy among the team of stakeholders, as the quality improvement team and the managerial team, has been reported as a positive factor to attain a timely achievement of goals and ensures an efficient implementation, adherent to plans.

Respondent 11 said:

‘Having the quality improvement team and facility management in our committee is good because when the team need a certain commodity these two teams push and something is done in time and ensure whatever we planned is executed accordingly’.

Involvement of the committee members not only in training activities but also in monitoring and correcting wrong practice was considered of help by Responder 05:

*‘…In our facility we conducted training on how to fill a partograph and we reviewed them every morning after clinical meeting so once we detect colleagues fill it badly we don't give off days’*.

### 4.3 Collective learning

Of interest for the reflecting and evaluating domain during the strategy implementation, collective learning has emerged as a central component of this construct. The systematic collection and sharing of data were considered central in regularly scheduled meetings involving different departments, to inform the practice and to modify strategies used to implement MDSR. Accordingly, the lack of the sharing opportunity as in regional meetings was perceived like a missed opportunity to improve the strategy through information exchange. Informant 01 said:

‘…We get these data from different departments in our health facility like labour ward, reproductive and child health unit, and pharmacy then we share this information in meetings about how many deliveries we have, how many referrals, referral feedback and staff have free chance to discuss and give opinions related to implementation. The place where it becomes difficult is when we participate in MDSR meeting at regional level: we don't get much time to discuss. But in our facility staff can learn from other staff’.

Knowledge sharing by members of the committee in community health educational events was considered essential to change the community attitudes. Respondent 05 said:

‘We provide health education to community members during outreach visits and with the pregnant mothers who visited our health facilities for antenatal clinic services we share the information regarding danger‐signs of pregnancies and need for early antenatal care attendance’.

The majority of participants considered the practice of continuing education in the healthcare facilities as related to the implementation of MDSR activities. Respondent 06 said:

‘We had a timetable of continuing education where colleagues prepare a session about a case and present it to us for teaching purposes. These sessions are attended by all staff in our facility so we use that time to teach each other based on the cases or scenarios’.

## 5 DISCUSSION

Our study has analysed the major implementation process factors that are considered to influence MDSR by MDSR committee's members in Dodoma City Council. According to our knowledge, this is the first study that has investigated aspects of the implementation of MDSR strategy in this setting.

Three major categories related to implementation processes emerged during interview of members of the MDSR committees: inclusiveness in the participatory planning process; stakeholders' readiness and accountability; and collective learning. This is in line with the construct for a learning climate defined by the CFIR as one in which leaders actively seek team members' inputs; team members, as essential partners in the change process, feel free and can try new methods; and members have opportunities for reflective thinking and evaluation.^19^

The study revealed that members of MDSR were involved in meetings scheduled to discuss the status of implementation of previous plans, and to formulate new ones accordingly. Participatory planning has been emphasized as one of the important components of the implementation processes, because it increases ownership of the intervention and shared responsibility. This is in line with what has been clearly expressed in a recently published scoping review by Kinney et al., on maternal and perinatal death surveillance and response in low‐ and middle‐income countries, in which the authors support the notion that the lack of ownership prevents effective implementation, while individual responsibility and ownership are essential to participation. A collaborative strategy among the team ensures the agreement among members, improves implementation monitoring, the building of a responsible delegation, finally leading to the effective continuity of the MDSR implementation processes.^20^ Additionally, the development of a culture of teamwork, with openness to new ideas, appreciation of differences and psychological safety, promotes shared insights and concerns, leading to learning.^19^

The involvement of managers has been perceived as a positive factor, catalyzing the resolution of issues and providing a timely provision of tools and satisfaction of needs towards an effective implementation. Leaders help to lay a bridge between the organization and its general and operating environments and promote and guide learning, by putting strategies into practice, through quality improvement and diffusion of information and knowledge.^19^ Engaged leaders have been widely recognized as implementation catalyzers, and studies are needed to understand what motivates leaders, what skills they should have and how to cultivate new champions.^20^

The engagement of appropriate stakeholders from multidisciplinary backgrounds and at different levels of the implementation process was perceived to increase their readiness and accountability in implementing the strategies, as when planning educational and engagement activities at the community level.

Data from Sierra Leone reported that stakeholders' involvement allows a direct confrontation with the reaching of targets, increases the readiness to support the implementation of MDSR activities in the community and it helps the user of the services to provide new ideas and inputs that could improve the process.^21^ Moreover, the study conducted by Adrian et al. (2016) in Sub‐Saharan Africa also reported that the involvement of individuals in the implementation process promotes individual accountability towards implementing the intervention^22^. Other studies emphasized the role of multilevel involvement of stakeholders in the prompt correction of mistakes, wherever a supportive supervision is put in place.^20^

The inclusion of different professional figures in the team has been considered as a positive element of the implementation process in previous studies.^22^ Multidisciplinary teams should include various cadres not only at the facility level, but also external stakeholders from ministries of health and other implementing partners at subnational and national levels.^20^ Because of cultural differences usually present within an organization across ranks, occupation, clinical specialties and operating units,^19^ in an environment favouring inclusion and confrontation a multidisciplinary team can act as a catalyzer for collective learning.

The virtuous engagement of staff and community healthcare workers together with the MDSR committee members in the provision of health education on maternal and infant death at the community level has been previously described in a pilot study conducted in South Africa. The authors emphasize the role of key stakeholders in gaining access to every part of the subdistrict covered by the intervention by maximizing resources and impact through coordinated, collaborative activities through the community network.^23^ This is in line with the social‐ecological perspective, a framework that recognizes the role of the community and local government and neighbourhood in determining health behaviour, interacting with individual and hospital level and healthcare organization, government and policy. This socio‐ecological perspective of the community role in health behaviour modelling can be extended to learning health systems, if we consider how ‘community’ factors exercise immediate influences on care practice.^19^

Collective learning has also been considered by respondents among the factors influencing the implementation of MDSR in healthcare facilities, allowing the improvement of the strategies in place according to individual input during planned discussions and presentation opportunities. This can be considered from the perspective of the organizational change framework, in which the individual learning must be communicated and managed for it to contribute to learning by other organization members, and teams can provide input into higher‐level learning while implementing evidence‐based practices.^19^ From this perspective, collective learning contributes to the organization‐level learning, by synthesizing learning at lower levels and applying the achieved knowledge to the pursuit of strategic priorities and organizational goals.^19^

It was noticed that the sharing of knowledge was easier at the council level but reduced at the regional level. This enhanced efficiency in sharing of knowledge at the lower level of the structure has been recognized in previous studies, which stated that subnational structures play a vital role in implementation.^20^

Current recommendations by WHO and UNICEF emphasize the importance of the use of data to inform practice to reach the goal of reducing maternal death,^20^ and highlight how knowledge sharing among pregnant mothers could also improve outcome, by increasing awareness.^24^, ^25^ According to a cross‐sectional study conducted in Zimbabwe on the evaluation of the national maternal mortality surveillance system, maternal death reviews are an important peer‐learning process that should remain central to the quality of care improvement strategy,^26^, ^27^ emphasizing the role of learning as a beneficial factor for an efficient implementation of MDSR.

### 5.1 Maternal death surveillance and response in a learning health system perspective

**FIGURE 2:**
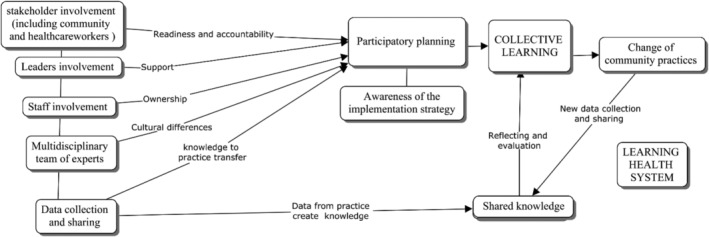
MDSR implementation network in a learning health system perspective.

Overall, the MDSR strategy can be defined as a tool to build, in the context of maternal health, a learning health system, as defined by the National Academy of Medicine as a system ‘in which science and informatics, patient‐clinician partnerships, incentives, and culture are aligned to promote and enable continuous and real‐time improvement in both the effectiveness and efficiency of care’.^19^ In this perspective, our findings emphasized the presence of several interactions among factors that respondents considered influential for MDSR implementation, within and across implementation levels, and through all the implementation network, that finally led to collective learning. As considered previously by Harrison and Shortell in the multilevel analysis of the learning health system, the internal and cross‐level alignment of these interacting factors included in the operating environment (communities, stakeholders, etc.) at the organization and mid‐management level (leadership), inside the team units (multidisciplinary teamwork) and at individual level (skills, knowledge, engagement) is at the base of the organizational learning^19^ that can close the knowledge to practice loop. This concept is summarized in Figure 2.

## 6 STUDY LIMITATION

This study was a qualitative study aimed at assessing the processes that were perceived to have an influence in the implementation of MDSR intervention in Dodoma city by members of the MDSR committee. Because of the study design, its results cannot be generalized to the whole population.

Differences across facilities in implementation of MDSR, which would have allowed to define the ‘level’ or ‘quality’ of implementation at various facilities, were not investigated in this study. Because of the small number of centres enrolled, exploring the difference among centres would not have been possible without a loss in confidentiality of each centre's respondents. While inductive content data analysis is a limitation for generalizability of findings, it has the advantage in the initial explorative phase of categories that may be studied with different methods in future.^28^

The potential bias due to fear of participants of the authoritative figure in the work institution was minimized ensuring confidentiality.

## 7 CONCLUSIONS AND RECOMMENDATIONS

The implementation processes perceived as influencing the MDSR intervention are the inclusiveness in participatory planning process, the stakeholders' readiness, support and accountability and collective learning.

In accordance with these findings, the Ministry of Health should conduct supportive supervision in all healthcare facilities in which MDSR is in place, to document and potentiate these aspects of the implementation processes and translate them into policies. Additionally, a precise stakeholder mapping could help increase their engagement.

Observational studies that focus on different levels of the implementation processes could be conducted in future using implementation frameworks.

## FUNDING INFORMATION

No funds were received to implement this project.

## CONFLICT OF INTEREST STATEMENT

The authors declare no conflicts of interest.
